# The Hairdex quality of life instrument—A translation and psychometric validation in patients with alopecia areata

**DOI:** 10.1002/ski2.220

**Published:** 2023-02-15

**Authors:** Johan Fhager, Åke Svensson, Karin Örmon, Tobias W. Fischer, Karin Sjöström

**Affiliations:** ^1^ Department of Care Science Malmö University Malmö Sweden; ^2^ Department of Dermatology and Venereology Lund University Skåne University Hospital Lund Sweden; ^3^ Department of Dermatology and Venereology Kepler University Hospital Johannes Kepler University Linz Austria

## Abstract

**Background:**

The German Hairdex quality of life (QoL) instrument is specific to hair and scalp diseases, developed for self‐rating and consists of 48 statements divided into five domains: Symptoms, Functioning, Emotions, Self‐confidence and Stigmatisation. There was a need of a Swedish reliability tested, validated hair and scalp specific QoL instrument why the German Hairdex was chosen to be translated and reliability tested in a systematic way.

**Objectives:**

To make a translation, a reliability test of stability, and validation of the German Hairdex QoL instrument among 100 Swedish patients with a dermatological ICD‐10 diagnosis of alopecia areata (AA).

**Methods:**

An eight‐step method by Gudmundsson was used as a model with a forward and backward translation and with comments from an expert panel. A statistical test–retest (ICC (2,1)) analysis was made, followed by an internal consistency analysis. A comparison between the German and Swedish Hairdex‐S constructs by a principal component analysis was performed.

**Results:**

The Hairdex‐S was very well accepted by patients. The ICC(2,1) test–retest showed a good to excellent correlation of 0.91 (CI [0.85–0.95]). Internal consistency was *α* = 0.92. Like the original Hairdex, Hairdex‐S showed good factorability with a Kaiser–Meyer–Olkin measure of 0.82 and with one component explaining 70% of the variance: original Hairdex instrument (69%). When tested on patients with AA, the domains Functioning and Emotions had the strongest loadings, followed by Stigmatisation and Self‐confidence. Younger AA patients at self‐assessment and patients who reported to be younger at the onset of AA, scored statistically significantly higher on the Hairdex‐S, indicating an overall lower QoL on domains Emotions and Functioning, respectively.

**Conclusions:**

The Hairdex‐S is very well accepted by AA patients, shows very good psychometric properties, and a very good agreement with the original Hairdex. The Swedish Hairdex instrument can be recommended for evaluation of patients QoL as well as for research purposes.

1

**What's already known about this topic?**

- QoL instruments are important tools in dermatology to reveal dysfunctions in mental health and social difficulties associated with the disease.
- Previous QoL instruments are not hair and scalp specific or enough validated. The German Hairdex instrument was developed for this purpose and was well‐accepted in patients with hair loss.
- The Hairdex is translated to other languages and used in studies of especially hair loss, but its reliability is yet not tested.

**What does this study add?**

- The Hairdex‐S has an excellent stability with a very high internal consistency and a similar construct as Hairdex. Hairdex‐S is well accepted by patients with alopecia areata. Younger age at disease onset and younger age at self‐assessment was related to lower QoL, especially emotions and functions.
- The use of Hairdex can be recommended for evaluation of patients quality of life as well as for research purposes.

## 2 INTRODUCTION

Assessments of quality of life (QoL) have become an integral part of dermatological studies and practice^1^, ^2^, ^3^, ^4^ and are important for estimating the burden of patients with skin conditions,^5^ QoL being a stronger predictor of psychiatric morbidity than the clinical severity per physician ratings.^6^ The burden of hair loss diseases such as alopecia areata (AA) and the current lack of efficient treatment, may cause vulnerability, such as lower self‐esteem and stigmatisation,^5^, ^6^, ^7^, ^8^, ^9^, ^10^, ^11^ which in turn may lead to psychological difficulties.^7^, ^8^, ^12^, ^13^

AA is one of the most common hair diseases and has a lifetime incidence of approximately 2% worldwide.^5^, ^9^, ^10^, ^11^ The disease is an inflammatory, non‐scarring hair loss disorder with an obscure aetiology with an autoimmune component.^9^ AA is characterised by hair loss in patches on the scalp that may proceed to a complete loss of hair on the scalp (alopecia totalis, AT) and further to a loss of all bodily hair (alopecia universalis, AU).^5^, ^9^, ^10^, ^11^

Negative changes in QoL are however found irrespective of visible hair loss.^14^ In view of the limited reporting of QoL in patients with hair loss, it is important to have a special self‐assessment instrument.^14^ The Hairdex self‐assessment QoL instrument published by Fischer et al.^14^, was validated in a sample of German women with either diffuse alopecia or androgenic alopecia. The Hairdex is a hair and scalp disease‐specific QoL instrument consisting of statements where patients can rate several QoL‐related problems.^14^ In addition, the Hairdex contains questions about loss of functioning, emotional experiences, stigmatisation and self‐confidence,^1^, ^14^ and it is used among patients with different kinds of hair loss.^5^, ^11^, ^15^, ^16^

Currently, there are no scientifically validated Swedish QoL instruments for hair loss and hair and scalp diseases which means that the QoL in this group of patients may not be noticed. Thus, a need exists for a translation and validation of the German Hairdex instrument. Since patients with AA are found to be especially vulnerable regarding stigmatisation^5^, ^9^, ^12^, ^17^ this group was considered appropriate for validation of the Swedish version of the Hairdex (Hairdex‐S). The aim of this study was to translate the German QoL instrument Hairdex into Swedish, as well as validating it in a Swedish AA sample.

## 3 MATERIAL AND METHODS

### 3.1 Construct of the original German Hairdex

The German Hairdex is based on the Skindex‐29^4^ four supplemented by two domains of Self‐confidence and Stigmatisation. The Hairdex statements are distributed over five QoL domains: Symptoms (eight items), Functioning (12 items), Emotions (11 items), Self‐confidence (nine items) and Stigmatisation (eight items).^14^ In the final Hairdex instrument, these 48 statements were changed by replacing skin by hair‐ and scalp‐related statements.^14^ The domain Functioning consists of QoL statements during work and leisure time and the domain Emotions contains statements about feelings of frustration and anger, and self‐reported symptoms of anxiety and depression caused by the hair and scalp condition. Different symptoms, such as burning sensation and pruritus, were included in the domain Symptoms, and the Self‐confidence domain contains statements regarding how confident patients feel despite their hair condition. Finally, the Stigmatisation domain contains statements about the perception and experience of other persons' attitude to the hair condition. A five‐item Likert scale, where the answer options are ‘0 = never, 1 = seldom, 2 = sometimes, 3 = often and 4 = always’ is used to rate each item.^14^

Six of the items in the Self‐confidence domain, numbers 31, 33, 35, 39, 41, 43 and number 40 in the Stigmatisation domain, are inverse and must be reversed and recomputed before analyses. Raw scores for the Hairdex range from 0 to 192 and are recommended by Fischer et al.^14^ to be linearly transformed to 0–100, higher values indicating lower QoL.

The Hairdex was validated in 75 women with either diffuse alopecia (*n* = 45) or androgenic alopecia (*n* = 30).^14^ The internal consistency of the different domains showed values with a Cronbach's *α* of 0.68–0.82, except for the Symptom subscale (*α* = 0.55), used as a reference.^14^ The instrument had good converging properties when validated against the Dermatological Life Quality Index (DLQI) and good discriminatory properties when validated against the Nottingham Health Profile tool (NHP).^14^ A principal component analysis showed one component explaining 69% of the variance. The primary test of the Hairdex showed that most women (78%) found the Hairdex easy to fill in and considered the questions relevant (90%) and understandable (69%). The average time for filling in the form was ≤15 min (70%) and ≤30 min (30%).

### 3.2 Translation of the Hairdex into Swedish (the Hairdex‐S)

After approval from the German authors, the translation of the Hairdex started, aiming to be as conceptually equivalent and as close to the German Hairdex as possible, inspired by the eight‐step translation model following Gudmundsson,^18^ with cultural differences taken into consideration.^19^ Step one to six and step eight in the Gudmundsson model were used. Instead of step seven, including pilot studies, we scrutinised the returned forms after the implementation of the translated instrument, with the purpose of identifying problems. In step one, the choice of a validated instrument, the Hairdex, was made. In step two and three, one translator and two members of an expert panel were recruited. The translating team had bilingual skills in German and Swedish and three had solid cultural context competence. In step four, a backward translation, with final comments by the panel was made. One translator, fluent in the German language, made the forward translation after reading the items several times. Subsequently, a Swedish‐born translator with German as her mother tongue, back translated the questions and commented on the translation in general. Finally, an independent panel of two German‐born Swedish physicians, one of them a dermatologist and one a specialist in infectious disorders both with German as their mother tongue and with excellent skills in Swedish, read both the German and the translated Swedish items and commented on the result and on any cultural expressional differences. In step five, the panel looked at differences between German and Swedish expressions in everyday language in an effort to examine equivalencies and resolve any possible discrepancies. Additionally, recommendations and answers when interpreting items was given by the German author of the original Hairdex. In step six, bias was considered, and in step eight, a validity test was performed. Two of the authors and the panel formulated the final version of the Hairdex‐S instrument.

### 3.3 Validation and reliability of the Hairdex‐S

The validation process took place at the dermatology outpatient unit, at the Skåne University Hospital in Malmö, Sweden. One hundred and two patients with AA were recruited. After written information 60 patients were collected from hospital registers, 30 patients by snowball effect and the remaining 12 patients from regular visits to the department's outpatient clinic. All patients gave their consent to be contacted by the same researcher and received complementary oral information about the study. Patients were interviewed between 12 December 2019 and 17 June 2021. Inclusion criteria were: AA diagnosis by a specialist in dermatology according to the ICD‐10,^20^ living in Scania, ≥18 years of age, and a good command of the Swedish language both orally and in writing. Exclusion criteria were: severe ongoing mental illness, severe ongoing abuse of drugs or intellectual disability. Two patients dropped out, one being advised to do so because of a severe mental condition, and one who found the study not in line with assumptions. The remaining patients (*N* = 100) fulfilled the inclusion criteria and signed a written consent of participation.

Before the self‐assessment of the Hairdex‐S, all patients were interviewed by the same interviewer. Information about medical and psychological conditions, sociodemographic data, and any presence of abuse, was obtained. The interviewer was present during the self‐assessments and could answer any questions. Two open‐ended questions were put forward by the interviewer at the end: ‘How did you feel when answering the Hairdex questionnaire?’ and ‘Did the questions describe what you have experienced?’ A reliability test–retest intraclass correlation coefficient (ICC) procedure with 30 patients randomly chosen out of the first 40 patients, was carried out. Information about the retest procedure was given and all patients gave their consent to participate. They received a pre‐stamped envelope containing the Hairdex‐S with a directive to fill in the form after 2 weeks. All patients returned the retest forms after a mean of two and a half weeks. One patient was reminded once.

### 3.4 Statistical analysis

Frequency analyses were used for descriptive sociodemographic variables. Correlations were made using Pearson product moment coefficient (*r*) for normally distributed data and Spearman rank sum coefficient (rho) for skewed data. Comparisons between groups were analysed with Mann–Whitney U test. Internal consistency was analysed with Cronbach's *α*. For the test–retest of stability, ICC (2,1) estimates and their 95% confident intervals were calculated using statistical package for social sciences (SPSS) statistical package version 28 based on a mean‐rating (*k* = 2), absolute‐agreement, 2‐way mixed‐effects model. For comparisons with the German Hairdex construction, a principal component analysis was made. A Kaiser–Meyer–Olkin measure for sampling adequacy (MSA) and Bartlett's Chi‐square were used for test of sampling adequacy and sphericity. Factor loadings, a scree plot and Eigenvalues were calculated. The SPSS version 28, (SPSS Inc., Chicago, IL) was used for all analyses.

### 3.5 Ethical considerations

The Swedish Ethical Review Authority approved the study, Reg.no. 2019–03811, which was conducted according to the ethical principles set out by the Helsinki Declaration.^21^ All participants were informed that they could decline participation at any time.

## 4 RESULTS

### 4.1 Content validation for the Hairdex‐S

An exact agreement of 96% between the Swedish and the German Hairdex instrument was found after translation. Two items (4%) were back translated with different meanings and were corrected after panel discussion. After the translation process, the panel made fine adjustments to improve sentences. The German ‘Meine Haare’ may be read as the quality of each hair or the hair as a whole, and the underlying meaning was adjusted for. Differences in German and Swedish items were mostly in wordings and not meanings, which indicates a high agreement between scales.

### 4.2 Reliability of the Hairdex‐S

Sociodemographic data for the AA‐sample are shown in Table 1. The mean (SD) for test and retest Hairdex‐S scores was 31 (13.8) and 27 (15.0), respectively. The ICC (2,1) analysis of stability showed a correlation of 0.91 with a confident interval = 0.85–0.95, which is seen as a good to excellent agreement. Internal consistency was high with an *α* = 0.92. Total score and mean value scores for the domains of the Hairdex‐S are shown in Table 2, together with the internal consistency for each domain, with self‐confidence indicating the greatest impairment of QoL (45.0) followed by emotions (36.5).Correlations between the domains of the Hairdex‐S are shown in Table 3. To compare the Swedish and the German Hairdex instruments, a principal component analysis was performed, which showed indicators of factorability to be good, with values of Kaiser‐Meyer‐Olkin = 0.82, and Bartlett's Chi‐square <0.0001, and low residuals indicated that the solution was a good one. Like the original Hairdex, one component was found with an Eigenvalue of ≥1, together with a scree plot confirming one component, which explained 70% of the variance in the Hairdex‐S compared to 69% in the original Hairdex. The component contained the Hairdex domains Functioning (0.93), Emotions (0.91), Self‐confidence (0.85), Stigmatisation (0.80) and Symptoms (0.67), the numbers representing the loading of each domain on the component. Since the construct validation of the two instruments showed very high similarities, no further analysis to compare convergent and discriminatory properties for the Hairdex‐S was needed.

**TABLE 1:** Demographic and biological data for the AA sample (*N* = 100).

Alopecia areata (AA)	
Alopecia areata, patch type (*n*)	60
Alopecia totalis/universalis (*n*)	40
Female (*n*)	90
Male (*n*)	10

Age at interview (years)	Mdn (range)	52.5 (18–93)
	mean (SD)	51 (16)
Age at onset of AA (years)	Mdn (range)	25 (0–78)
	mean (SD)	28 (17.3)
Duration of AA (years)	Mdn (range)	17.5 (1–71)
	mean (SD)	22 (17)

**TABLE 2:** Scale parameters of the five domains of the Hairdex‐S for the AA sample (*N* = 100).

Domain scores	Mean	SEM	Median	SD	Skewness	Kurtosis	Cronbach's *α*
Symptoms	22.0	1.99	19	19.89	1.00	0.56	0.82
Functioning	28.0	2.33	23	23.33	0.96	0.58	0.92
Emotions	36.5	2.70	33	26.96	0.49	0.64	0.94
Self‐confidence	45.0	1.91	42	19.10	0.53	0.31	0.66
Stigmatisation	27.8	1.91	25	9.14	0.78	0.90	0.76
Total score	32.0	1.89	30	18.87	0.83	0.60	0.89

*Note*: The Hairdex‐S: Swedish translation of the Hairdex.

**TABLE 3:** Correlation matrix for scores of the five domains in the Hairdex‐S (*N* = 100).

Domains	Symptoms	Functioning	Emotions	Self‐confidence	Stigmatisation
Symptoms	1.00	0.54	0.62**	0.41**	0.34**
Functioning	0.54**	1.00	0.85**	0.74**	0.69**
Emotions	0.62**	0.85**	1.00	0.68**	0.59**
Self‐confidence	0.41**	0.74**	0.68**	1.00	−0.69**
Stigmatisation	0.34**	0.69**	0.60**	−0.69**	1.00

*Note*: The Hairdex‐S: Swedish translation of the Hairdex. Correlations analysed with the Pearson's product moment correlation test.

*p < 0.05; **p < 0.001.

### 4.3 Validation of the Hairdex‐S in an AA sample

Most patients (96%) found the items relevant and easy to answer. Some (4%) had problems filling in the items due to old age or dyslexia. All statements were told to capture the patients experiences of AA. Items from the domains Self‐confidence and Stigmatisation were considered particularly valuable. Most patients filled in the form in ≤15 min. Patients who had suffered from AA for several years, experienced some questions as better suited for the newly diagnosed and those who suffered from AT or AU found items about hair loss not relevant. The last item, number 48, about ‘being taken seriously by their doctor’, was often commented by patients as very important, especially during the diagnostic process of AA.

Biodemographic data for the sample are shown in Table 1. Most patients suffered from patches (60%). Male patients (10%) were statistically significantly younger than female patients (41 [20.0] vs. 52 [15.4]; mean [SD]; *p* < 0.04). No other statistically significant differences were found between men and women with respect to total score and domain scores of the Hairdex‐S, or with respect to the duration or age at onset of AA.

Domain values are shown in Table 2. The highest mean domain values among patients were found for Self‐confidence followed by Emotions, higher values meaning lower QoL. The highest correlations found were between Functioning and Emotions (rho = 0.85) and between Functioning and Self‐confidence (rho = 0.74), both *p* < 0.001 (Table 3).

At the self‐assessment, younger patients rated their total QoL lower (rho = −0.31; *p* < 0.002) and their QoL lower in the domains Emotions (rho = −0.34), Symptoms (rho = −0.29) and Functioning (rho = −0.26); all *p* < 0.01. Lower total QoL was also rated by patients who reported to be younger at disease onset (rho = −0.30; *p* < 0.001), with lower QoL ratings in the domains Functioning (rho = −0.38; *p* < 0.0001), Self‐confidence (rho = −0.28; *p* = 0.03) and Emotions (rho = −0.22; *p* = 0.005).

## 5 DISCUSSION

The Hairdex‐S was found to be well accepted by patients with AA. Hairdex‐S showed a very high stability and internal consistency. The German and Swedish patients' positive perception of the instrument and the similarities in constructs of both instruments increases the validity. The translation was easy to perform, and the German and Swedish instruments seem to have a high common face and content validity. Younger patients at disease onset reported lower QoL in the domains Emotion and Functioning. To our knowledge, this is the first time a Hairdex instrument's reliability is tested, which means that a comparison regarding reliability is undoable.

Several methods are recommended regarding the translation of instruments, such as the WHO Guidelines on Translation and their ITC Guidelines for Translating and Adapting Tests.^19^ We chose to use the eight‐step translation procedure inspired by Gudmundsson^18^ since it was structured and easy to follow. It was a strength that three members of the translating team had knowledge in both German language and German culture as well as knowledge about Swedish language and culture. This kind of knowledge has been considered especially important when translating QoL instruments.^22^

We found almost the same results for the QoL domain score values as were found in the original Hairdex.^14^ Differences between German and Swedish study populations may explain why scores for the domain Self‐confidence were considerably higher in our study indicating a lower QoL. The Swedish sample was younger, of mixed gender, with a wider age range and contained patients with AA exclusively, whereas the German female sample was somewhat older and suffered from both diffuse alopecia and androgenetic alopecia. Furthermore, the Swedish sample contained patients with more severe AA, diagnosed by the ICD‐10.^20^ Younger patients with more severe AA may assess their QoL lower in domains such as Self‐confidence. Compared to the German Hairdex study for more severe AA, that is, obvious hair loss, we found somewhat better QoL regarding Symptoms and lower QoL regarding Stigmatisation, which may be due to both illness severity, age and cultural variations. In both German and Swedish Hairdex instruments, the lowest QoL estimation was found for domains Self‐confidence and Emotions and the highest for domain Symptoms. This could be explained by the impact of hair loss on self‐esteem and the emotional reactions of anger, anxiety and depression. A higher QoL estimation was found for domain Symptoms in both instruments, which may be explained by the relatively lower impact that pain and other symptoms have in hair loss diseases. The difference in patients diagnoses between the German and the Swedish samples may explain the slight differences on QoL domain estimation.

A Turkish and an English version of the Hairdex already exist.^13^, ^15^ The English Hairdex version was an in‐house translation made without reliability testing.^15^ The Turkish version was translated without reliability testing, but the Hairdex scores were compared to a Turkish version of a QoL instrument.^13^ All patients in the translated versions of Hairdex have found the instrument appealing, and so did patients in the present study.

The original Hairdex^14^ and the English version^15^ of the instrument differed in one aspect, namely, item 47, the phrasing ‘…that the patient looked in the mirror to see if the hair had become sparser’ being translated in the English version to ‘if there was any change of my hair’. Another discrepancy that researchers must be aware of when translating from the English Hairdex is item 3, which is translated to ‘I worry about my hair loss…’ when the original German item says, ‘I worry that there is a serious problem with my hair’. This is important since the Hairdex is an instrument that focuses on both hair loss and other diseases of the hair. In the Swedish version, a printing error was found in item 25 in the domain Functioning, but after careful post‐analyses of the item within the domain and of the item score related to the total score, this error was not considered to change the result and was later corrected in the Hairdex‐S form. Another important methodological issue when comparing studies using the Hairdex, is if a statement regarding reversal of scores has been made. If not, there is a risk that both the total Hairdex scores and the Self‐confidence and Stigmatisation domain scores is not appropriately interpreted.

In a Turkish study, by Gonul et al.,^13^ on the severity of hair loss and QoL measured by Hairdex, they found lower QoL with increasing disease severity regarding domains Emotions, Functioning, Stigmatisation, Self‐confidence and total Hairdex score. In the present study, we could not confirm these results when comparing AA severity.

Several attempts have previously been made to create QoL instruments specific to AA.^23^, ^24^, ^25^ However, those instruments have not yet been used in clinical studies, which makes it difficult to draw any conclusions about their importance in clinical AA populations. Chernyshov et al.^5^ suggest that further extensive validation is needed for all the AA‐specific instruments.

There is a growing scientific interest in AA and the impact on QoL since new therapies are about to be introduced. Chernyshov et al.^5^ suggest that new treatment options, for example, immune therapy, may lead to an increased interest in using reliable and sensitive instruments to assess health‐related QoL in patients with AA. The result of this study showed that AA has a negative impact on QoL as measured by the Hairdex mainly in areas of self‐confidence and emotions, which may give rise to longstanding effects, especially if patients have been suffering from a young age. We agree with Schielein et al.,^11^ who stated that further studies are needed in order to better comprehend the psychosocial burden of hair loss. The reliability confirmation and validation of the Hairdex‐S will improve information on hair diseases and hair loss. The instrument can be useful both in research and clinically as a tool for assessment of QoL when visiting the dermatologist.

## 6 CONCLUSION

- The Hairdex‐S instrument has good to excellent validity and reliability properties.
- The Hairdex‐S instrument is easy to administrate and is well tolerated among patients since it is easy to fill in, though older patients may need more time to capture the questionnaire statements.
- The Hairdex‐S instrument may be seen as an estimate of the effect of the hair and scalp condition on QoL. It may hence be recommended as a preparation before visiting the dermatologist as well as providing information when clinically deciding on new treatments.

## CONFLICT OF INTEREST STATEMENT

None to declare.

## AUTHOR CONTRIBUTIONS

**Johan Fhager**: Conceptualization (equal); Data curation (equal); Formal analysis (equal); Investigation (equal); Methodology (equal); Project administration (equal); Supervision (equal); Validation (equal); Writing – original draft (lead); Writing – review and editing (equal). **Karin Ormon**: Conceptualization (supporting); Data curation (supporting); Formal analysis (supporting); Investigation (supporting); Methodology (supporting); Supervision (supporting); Validation (supporting); Visualization (supporting); Writing – original draft (supporting); Writing – review and editing (supporting). **Tobias W. Fischer**: Methodology (supporting); Supervision (supporting); Visualization (supporting); Writing – review and editing (supporting). **Åke Svensson**: Data curation (supporting); Formal analysis (supporting); Funding acquisition (supporting); Investigation (supporting); Methodology (supporting); Resources (supporting); Supervision (supporting); Validation (supporting); Visualization (supporting); Writing – original draft (supporting); Writing – review and editing (supporting). **Karin Sjostrom**: Conceptualization (equal); Formal analysis (equal); Investigation (equal); Methodology (equal); Project administration (equal); Resources (equal); Supervision (equal); Validation (equal); Visualization (equal); Writing – original draft (equal); Writing – review and editing (equal).

## ETHICS STATEMENT

The Swedish Ethical Review Authority approved the study (Reg.no. 2019–03811).

## Data Availability

Data that support the findings of this study are available from the corresponding author upon reasonable request and with ethical considerations.
